# Curtailing virus-induced inflammation in respiratory infections: emerging strategies for therapeutic interventions

**DOI:** 10.3389/fphar.2023.1087850

**Published:** 2023-05-05

**Authors:** Alexander A. Globenko, Gennady V. Kuzin, Anastasia V. Rydlovskaya, Elena I. Isaeva, Elizaveta N. Vetrova, Tat’yana N. Pritchina, Ancha Baranova, Vladimir E. Nebolsin

**Affiliations:** ^1^ JSC Valenta Pharm, Moscow, Russia; ^2^ RSV Therapeutics LLC, Moscow, Russia; ^3^ N F Gamaleya Federal Research Center for Epidemiology & Microbiology, Moscow, Russia; ^4^ School of Systems Biology, George Mason University, Fairfax, VA, United States; ^5^ Research Centre for Medical Genetics, Moscow, Russia; ^6^ Pharmenterprises LLC, Moscow, Russia

**Keywords:** virus-induced lung inflammation, host response approach, hyperinflammation, antiviral therapy, cytokine storm, anti-inflammatory, chemokine

## Abstract

Acute respiratory viral infections (ARVI) are the most common illnesses worldwide. In some instances, mild cases of ARVI progress to hyperinflammatory responses, which are damaging to pulmonary tissue and requiring intensive care. Here we summarize available information on preclinical and clinical effects of XC221GI (1-[2-(1-methyl imidazole-4-yl)-ethyl]perhydroazin-2,6-dione), an oral drug with a favorable safety profile that has been tested in animal models of influenza, respiratory syncytial virus, highly pathogenic coronavirus strains and other acute viral upper respiratory infections. XC221GI is capable of controlling IFN-gamma-driven inflammation as it is evident from the suppression of the production of soluble cytokines and chemokines, including IL-6, IL-8, CXCL10, CXCL9 and CXCL11 as well as a decrease in migration of neutrophils into the pulmonary tissue. An excellent safety profile of XC221GI, which is not metabolized by the liver, and its significant anti-inflammatory effects indicate utility of this compound in abating conversion of ambulatory cases of respiratory infections into the cases with aggravated presentation that require hospitalization. This drug is especially useful when rapid molecular assays determining viral species are impractical, or when direct antiviral drugs are not available. Moreover, XC221GI may be combined with direct antiviral drugs to enhance their therapeutic effects.

## Introduction

Acute respiratory viral infections (ARVI) are the most common illnesses worldwide. A majority of the viruses affecting the human respiratory tract have their proteins encoded in RNA. Most concerning viral species are influenza viruses, respiratory syncytial virus (RSV), rhinoviruses and coronaviruses (CoVs), with RSV causing the most severe lung pathology (bronchiolitis) in children in their first year of life, with high mortality rates (Paes et al., 2016). In later ages, the damage caused by RSV and rhinoviruses is associated with an increased risk of asthma (Bergroth et al., 2020). Another virus of concern is influenza type A. With every novel subtype of pandemic influenza, there is a rise in the severity of clinical symptoms of the flu (Macias et al., 2021; Paget et al., 2019). Recently, a continued pandemic of SARS-CoV-2 has been added to the seasonal coronaviruses (Johns Hopkins Coronavirus Resource Center 2022).

ARVI-causing viruses are recognized by pattern recognition receptors (PRRs). Specifically, the presence of RNA viruses is sensed by Toll-like receptors (toll-like receptors, TLRs) 2, 3, 7, 8 and cytosolic RIG-like and NOD-like receptors (Deliyannis et al., 2021; Khan et al., 2021). After entering the cell, the virus triggers a massive release of proinflammatory cytokines (Schultze and Aschenbrenner, 2021), including interferons (IFN) I (α, β) and III (λ) types. These molecules signal to neighbouring uninfected epithelial cells, thus, promoting costly intracellular response, which, in turn, halts replication of viruses. Meanwhile, type II (γ) IFNs promote the release of chemokines by engaging CXCR3. If the interferon-guided suppression of the viral reproduction is not efficient, immune cells ramp up the secretion of immune cell-directed proinflammatory cytokines (IL6, TNFα), which summon the neutrophils and the T cells into the respiratory tract, thereby, promoting the local development of unbalanced, massive inflammatory assault [Vardhana and Wolchok, 2020; Shimabukuro-Vornhagen et al., 2018; Harker et al., 2011].

This type of hyperactive inflammatory response is known as “cytokine storm” and may be caused by any ARVI. Hyperinflammation is especially common in the lungs of patients with primary or secondary immune imbalances or with disruption of adaptive immunity activation. Excessive pulmonary inflammation may causally contribute to acute respiratory distress syndrome and increase the chance of acquisition of secondary bacterial infections (Wu et al., 2022; Nye et al., 2016). When hyperinflammation involves the blood vessels–as it commonly occurs in COVID-19—the dysfunction of coagulation follows (Len et al., 2022), and leads to a host of body-wide complications.

Both in the COVID-19 pandemic and in the seasonal ARVI outbreaks, physicians’ offices see a rising tide of moderate-to-severe cases with recognizable hyperinflammation and a significant intoxication syndrome accompanied by chills, fever, sudden onset headache, distant aches, dizziness, nausea, vomiting and fatigue. In case when specific viral infection is diagnosed, either direct or antibody-based antivirals may be used, including oseltamivir, zanamivir, baloxavir, ribavirin, palivizumab, nirmatrelvir with ritonavir, remdesivir, or molnupiravir. These treatments prevent the disease from taking a severe course, and help the patient to avoid secondary complications (Tesini, 2022). In other words, early treatment with antivirals halts the development of a virus-driven hyperinflammatory condition and resultant intoxication syndrome (Lansbury et al., 2019; Chen et al., 2020; Kositpantawong et al., 2021). Above mentioned scenario is highly desirable, but quite uncommon as real world data show the low utilization, and the low rates of timely administration of antivirals, predominantly due to the challenges of molecular diagnostics at the point-of-care encountered (Lee J. et al., 2019). Even in patient’s cohort with confirmed influenza, utilization of antiviral treatments remains suboptimal (32.5% Tse et al., 2022; 33.5% Chen et al., 2020). In turn, widespread under-utilization of direct antivirals leads to an excessive need in hospitalization of hyperinflammatory cases.

On top of this, non-influenza ARVIs, which are typically mild when observed in general population, may produce hyperinflammation in vulnerable individuals, and, therefore, contribute to seasonal peaks of hospitalizations with severe respiratory inflammation [Nair et al., 2010; Peteranderl et al., 2016; Walsh and Hall, 2015]. In the multiyear Canadian surveillance cohort study of more than 2000 hospitalized patients, many were admitted with severe non-influenza ARVI (human rhinovirus 14,9%; RSV 12,9%; non-COVID-19 coronavirus 8.2%) and 45,4% had severe influenza (Lee N. et al., 2021).

Therapeutic strategies suitable for patients with viral respiratory infections are a subject of heated discussions. Apparent advantage of direct antiviral agents (DAAs) is their ability to promote the viral eradication. Because of that, DAAs represent a large part of the world’s pipeline of drugs for ARVIs. However, approved ARVI’s DAAs control influenza and SARS-CoV-2, while non-influenza, non-SARS-CoV-2 ARVI remained not yet targeted. Moreover, DAAs are not free from serious disadvantages, including the distinct possibility for selecting the treatment-resistant viral subtypes, which is already reported for oseltamivir, baloxavir marboxil and nirmatelvir (van Schalkwyk, 2021). Therefore, the therapies aimed at curtailing the virus-induced hyperinflammatory condition in ARVIs are both applicable to current real-world situation and highly sought.

The prevalence of hyperinflammation varies, depending on the type of virus. In general population, the largest rates are seen in patients with COVID-19 and pandemic influenza A, including 2009 H1N1 and 2013 H7N9 strains. In all these infections, the activation of lung endothelium and the dysfunction of coagulation are commonly seen. In high-risk group which includes infants and elderly patients with severe seasonal ARVI (CDC: RSV Centers for disease control and prevention, 2022), the burden of hyperinflammation is also high. For example, among children of 5 years and under, a total of 4.8 million hospitalizations due to RSV recorded annually (Nair et al., 2010). In population aged 60 years or more, active surveillance of post-rhinovirus infections led to uncovering community-acquired pneumonia illness in up to 28% follow-ups. As a result, approximately a quarter of this cohort lost their ability to perform routine household activities, and nearly 20% were confined to bed (Jain et al., 2015).

Here we describe available preclinical and clinical data on XC221GI. This molecule was identified in a screen of imidazolyl ethanamide pentandioic acid (IEPA) scaffold derivatives (Malik et al., 2020). XC221GI has yielded a top result in a screening of novel chemicals performed in cotton rats infected with RSV, and later, in 2017, was found to reduce viral load in the lungs of golden hamster after oral inoculation with the SARS-CoV-1 virus.

An exhaustive round of preclinical studies, including assessments of animal survival rate, body weight, and viral load dynamics, confirmed XC221GI as valuable candidate for a preventive anti-inflammatory therapy in model infections with influenza, RSV, SARS-CoV-1, rhinovirus, human paramyxovirus, and human metapneumovirus.

### Preclinical development and pharmacological effects of XC221GI

Below we describe the results of preclinical studies performed in 2014–2019 with the viruses commonly causing ARVIs. As could be seen from the described below, initial studies were aimed at studying direct antiviral effects of XC221GI. These results (not published) were certainly interesting, but shed a little light on the mechanisms of antiviral action of XC221GI.

#### H3N2 influenza virus

The H3N2 influenza virus is one of the most common among circulating strains every year (Paules and Subbarao, 2017). Therefore, we selected the A/Aichi/2/68 H3N2 influenza strain, a gold standard for respiratory tract inflammation studies, as a model of infectious agent for seasonal influenza model in C57Bl/6 mice.

Effects of XC221GI on cells infected with influenza virus strain A/Aichi/2/68 (H3N2) were studied in male C57 BL/6 mice (age 3–4 weeks, body weight 11–14 g). In first experiment, XC221GI was administered at a dose of 15 mg/kg/day for 5 days following influenza virus infection at a dose of 3.2 TCID 50/ml via gavage. In this study, indicators of viral activity at 4, 5, and 6 days after infection were substantially lower than that in control animals (*p* < 0.05 for all observations). Notably, in influenza virus-infected male C57BL/6 mice treated with XC221GI at a dose of 30 mg/kg/day for 3 days, there was a statistically significant decrease in mortality (*p* < 0.05), which amounted to 20% of observed events as compared to 62% in a control group. In the second experiment, performed in animals infected with 5.2 TCID 50/ml, a post-infection XC221GI dose of 30 mg/kg was administered via gavage either once or once a day for 3 days. In both arms of this experiment, at the Day 4, animals treated with XC221GI lost lower percentage of body weight than control group. In both studies, animal were sacrificed, and the lung homogenates collected. In treated groups, a pronounced decrease in the viral titres and viral RNA copy number at 96 and 120 h after infection was significant and dose dependent. In third experiment, C57Bl/6 mice were infected with 5.2 50% Embryo Infectious Dose (EID50). Animals in an experimental group were treated with XC221GI three times a day at time points of 45 h, 69 h and 93 h after infection at a dose of 15 mg/kg or 30 mg/kg. A pronounced decrease in viral titer and the number of copies of virus RNA was observed in lung homogenates taken 96 and 120 h after infection.

#### Human respiratory syncytial virus (RSV)

In RSV infection, effects of XC221GI were evaluated in 3–4 weeks old male BALB/c mice. Intranasal challenge with KP713401 RSV was performed at a dose of 5.0 lg TCID 50/ml. Three regimens were tested. Arm 1: at the time point of 24 h post-infection, XC221GI at a dose of 30 mg/kg for 5 days. Arm 2: similar to Arms 1, but with 7 days of treatment. Arm 3: at the time point of 72 h post-infection at a dose of 30 mg/kg/day for 5 days. In this study, a comparison group was treated with ribavirin at a dose of 40 mg/kg/day for 5 days. Additionally, a group of intact animals (negative control), and a group of infected animals that did not receive any treatment (positive control) were included.

In Arm 1, Arm 2, and Arm 3 on Days 7 and 9 after infection the average lung weight was lower than that in positive control group, indication lesser extent of edema and inflammation (*p* < 0.05 at both time points). In a treatment comparator group (ribavirin), no statistically significant differences in the body weight of animals or weights of their lungs when compared to positive control group were noted (*p* > 0.05).

In Arm 2, on Day 9, average body weight of mice treated with XC221GI was higher than that in positive control group (*p* < 0.05). Additionally, on Days 5, 7 and 9, viral titres in lung homogenates were lower (*p* < 0.05). In Arm 1, where mice have received a shorter 5 days treatment, the differences in viral titres were notable only on days 5 and 7 of observation (*p* < 0.05), indicating possible short-term rebound. On the ninth day of observations, a histopathological analysis of the lungs collected from animals of Arm 1, Arm 2 and Arm 3 confirmed a significant point-scale decrease in involvement of pulmonary tissues (*p* < 0.05 vs. with the positive control group).

In ribavirin-treated comparator group viral titers and RSV RNA levels were found similar to that observed in Arm 2.

Notably, in both XC221GI treated arms and in ribavirin-treated comparator group, the levels of T-cell and NK-cell stimulatory cytokine IL-12 were measured on Days 1, 3 and 5 and found similar, while being higher than in untreated mice (*p* < 0.05).

#### Rhinovirus

The activity of XC221GI against human rhinovirus (viral strain 2,730) was investigated in male BALB/c mice in three Arms, namely, Arm 1: XC221GI gavage at a dose of 3 mg/kg/day; Arm 2: 15 mg/kg/day; Arm 3: 30 mg/kg/day. A comparator Arm was treated with ribavirin at a dose of 40 mg/kg/day. In all Arms, animals were treated for 4 days.

In Arm 2, decreases in viral titres in the lung homogenates were amounting to 1.7 and 1.8 lg TCID50/ml on days 2 and 4, respectively (*p* < 0.05 when compared to positive control), and were commensurate with that observed in the ribavirin group. Similar significant changes were observed for viral RNA copy numbers at a XC221GI dose of 3 or 15 mg/kg/day or ribavirin at a dose of 40 mg/kg/day. Notably, in highest XC221GI dose of 30 mg/kg/day (Arm 3), respective decreases in viral titres and the copies of viral RNA were significantly different from the control only at a Day 4.

#### Human paramyxovirus

In the study of XC221GI activity against SeV Sendai virus MPMV1 infected male outbred mice (n = 120, body weight 10–12 g, age 3–4 weeks), oral gavage treatments were administered 24 h after infection at 3, 15, and 30 mg/kg/day for 5 days, with a comparator arm treated with ribavirin at a dose of 40 mg/kg/day.

In this study, no effects on the body weight loss in infected animals were detected in any of the treatment Arms, including one with ribavirin (*p* > 0.05). However, in the survival analysis, molnupiravir (Kaore and Atal, 2022) and nirmnatelvir/ritonavir combination (Akinosoglou et al., 2022) administration of compound XC221GI at doses of 3 or 15 mg/kg/day was accompanied by a statistically significant decrease in mortality by 30% and 45%, respectively (*p* < 0.05).

#### Human metapneumovirus

In male BALB/c mice (n = 100, body weight 10–12 g, age 3–4 weeks) infected with human metapneumovirus HPMV through nasal cavities, effects of oral gavages of XC221GI at a dose of 15 or 30 mg/kg/day or ribavirin at a dose of 40 mg/kg/day were studied for 7 days.

In a 30 mg/kg/day dose arm, the survival rate was at 75%, which was comparable to that in the ribavirin group and significantly higher than the positive control group, where the survival rate not exceeded 35% (*p* < 0.05). In a dose of 15 mg/kg/day arm, the survival rate of animals was at 60%, and not significantly different with that in positive control group.

**FIGURE 1:**
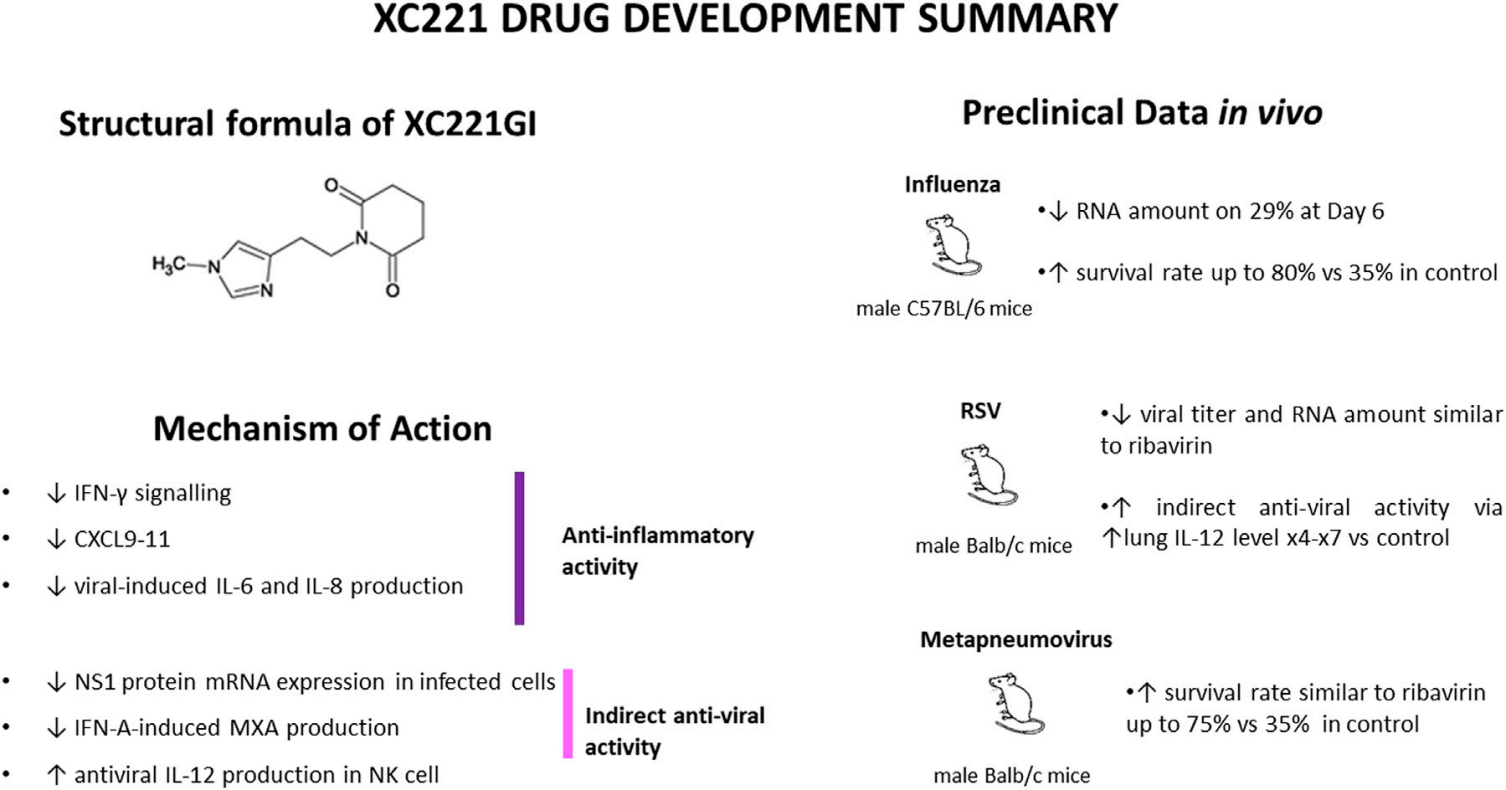
XC221GI drug development summary.

The results of these studies are summarized on Figure 1 and Table 1.

**TABLE 1:** Specific effects of XC221GI in various viral infections and its mechanisms of action.

Virus type	Specific effects of XC221GI	Mechanisms of action
Influenza H3N2	↓ viral titer x2 vs control (*in vivo*)	↓ IFN-γ signalling by 20–25%
	↓ viral RNA amount on 29% at Day 6 (*in vivo*)	
	↑ mice survival rate up to 80% vs 35% in control (*in vivo*)	
RSV	↓ viral titer and RNA amount similar to ribavirin (*in vivo*)	
	↓ RSV-induced IL-6 and IL-8 production by A549 cell (*in vitro*)	
	↓ NS1 protein mRNA expression in respiratory epithelial cells	
	A549 (*in vitro*)	↓CXCL9, CXCL10, CXCL11 production by monocytes, macrophages, endothelial cells and peripheral blood mononuclear cells
SARS-CoV-1	↓ viral titer at day 2 in preventive protocol (*in vivo*)	
	↓ viral titer at day 2 & day 6 in treatment protocol (*in vivo*)	
Sendai virus	↓ mice mortality rate up to 45% (*in vivo*) (in vivo)	
Metapneumovirus	↑ survival rate similar to ribavirin up to 75% vs 35% in control (*in vivo*)	
Human rhinovirus	↓ viral titer similar to ribavirin (*in vivo*)	

### Anti-inflammatory effects of XC221GI in the *in vitro* and *in vivo* models of ARVIs

In uninfected A549 cell culture, XC221GI did not affect the levels of IL-8 secretion, while the levels of IL-6 secretion are below the limits of detection even at the baseline. When the A549 cell culture infected with RSV were exposed to XC221GI for 48 h, the secretion levels of IL6 and IL-8 were down by 55%–85% by 40%, respectively. A 50% of IL6 inhibition with XC221GI was achieved at 27.6 nmol/L (6.1 ng/mL) (Stukova et al., 2022). Therefore, the next series of experiment was directed at assessing the anti-inflammatory activity of XC221GI in RSV-induced lung pathology in cotton rats [Boukhvalova et al., 2018; Blanco et al., 2014].

The treatments have started 24 h after infection at a dose of 20 mg/kg/day for 5 days with drinking water. At the Day 6, when lungs were removed for histopathological evaluation, a significant decrease in the severity of alveolitis and interstitial pneumonia was notable when XC221GI treated group was compared to a control one.

In an immunosuppressed cotton rat model, the maximal scores of lung pathology are observed at Day 10 post-RSV infection. In this model, the severity of interstitial inflammatory cell infiltration in XC221GI-treated cotton rats was also lower than that in placebo-treated animals. The same model was utilized for a study of XC221GI at a dose of 15 mg/kg which was administered 8–12 h after intranasal exposure to IFNγ. A significant decrease in the number of neutrophils was noted in XC221GI treated group when compared to the vehicle-treated animals. In this model, the neutrophil-lowering effects of XC221GI were comparable to that of dexamethasone (1 mg/kg). A single administration of XC221GI at doses of 3 and 15 mg/kg significantly reduced the levels of CXCL10 and CXCL9 chemokines followed the administration of IFNγ. In dexamethasone-treated group, the levels of CXCL9, but not CXCL10 were lowered. At a dose of 15 mg/kg, treatment with XC221GI also led to a significant decrease in the levels of CXCL11 in bronchoalveolar lavage at a time point of 8 h post-IFNγ exposure (Stukova et al., 2022).

The results of the XC221GI–related preclinical research are summarized in Table 1.

### Results of a preclinical toxicological study *in vivo*

In mice and cotton rats treated with escalating doses of XC221GI there were no macroscopic or histological defects of any tissues. In particular, the gastric mucosa tissue was thoroughly assessed, and no damage found. In a panel of standard *in vitro* and *in vivo* tests, including Ames test, test for the induction of chromosomal aberrations, micronucleus test, and alkaline elution test, no cytotoxic, mutagenic, genotoxic, or carcinogenic potential was detected in a wide range of doses (up to 5,000 mg/kg). Moreover, XC221GI is not metabolized by the liver and, therefore, does not interact with the human cytochrome system.

A study of reproductive toxicity performed in adult male and female rats, application of XC221GI in a daily dose of 17.4 or 174 mg/kg, an equivalent of one and ten therapeutic doses in humans, led to no changes in the fertility index or patterns or percentage of the post-implantation death. No fetal, prenatal or postnatal defects were observed (Suhanova et al., 2021).

The study of immune responses did not reveal any adverse effects of XC221GI on the antibody formation, the number of T- and B-lymphocytes or phagocyte activity. No delayed or immediate hypersensitivity reactions were detected in the studied animals in doses up to ten therapeutic equivalents. XC221GI exerted no influence on the blood cells, the hematopoiesis or the hemostasis. The dose of no apparent adverse effect (NOAEL) was 30 mg/kg per day for dogs and 450 mg/kg per day for rats (Suhanova et al., 2021).

In conclusion, the study of acute and chronic toxicity of XC221GI was performed in five animal species, including mice, rats, guinea pigs, rabbits, and dogs) and showed that it is tolerated well, with no significant changes in the behaviour of animals, in the consumption of feed and water. No animal was observed throughout the periods of dosing and recovery. *In vivo* experiments showed no signs of toxicity or other undesirable effects after a single administration of XC221GI to outbred mice and rats of both sexes at a dose of 5,000 mg/kg, as well as after repeated administration to rats, rabbits, and dogs of both sexes in studies with 2 weeks, 4 weeks, 30-day and 3-month dosing of XC221GI at the doses of 6–174 mg/kg per day, which, after taking into account interspecies conversion factors, corresponds to the range of human therapeutic doses of one to ten, with proposed daily therapeutic dose in humans being 200 mg (Suhanova et al., 2021).

Based on the favourable metabolic safety profile and its anti-cytokine effects, XC221 was advanced to the testing in humans under the trade name Aterixen^®^.

### Clinical trials of XC221GI

**FIGURE 2:**
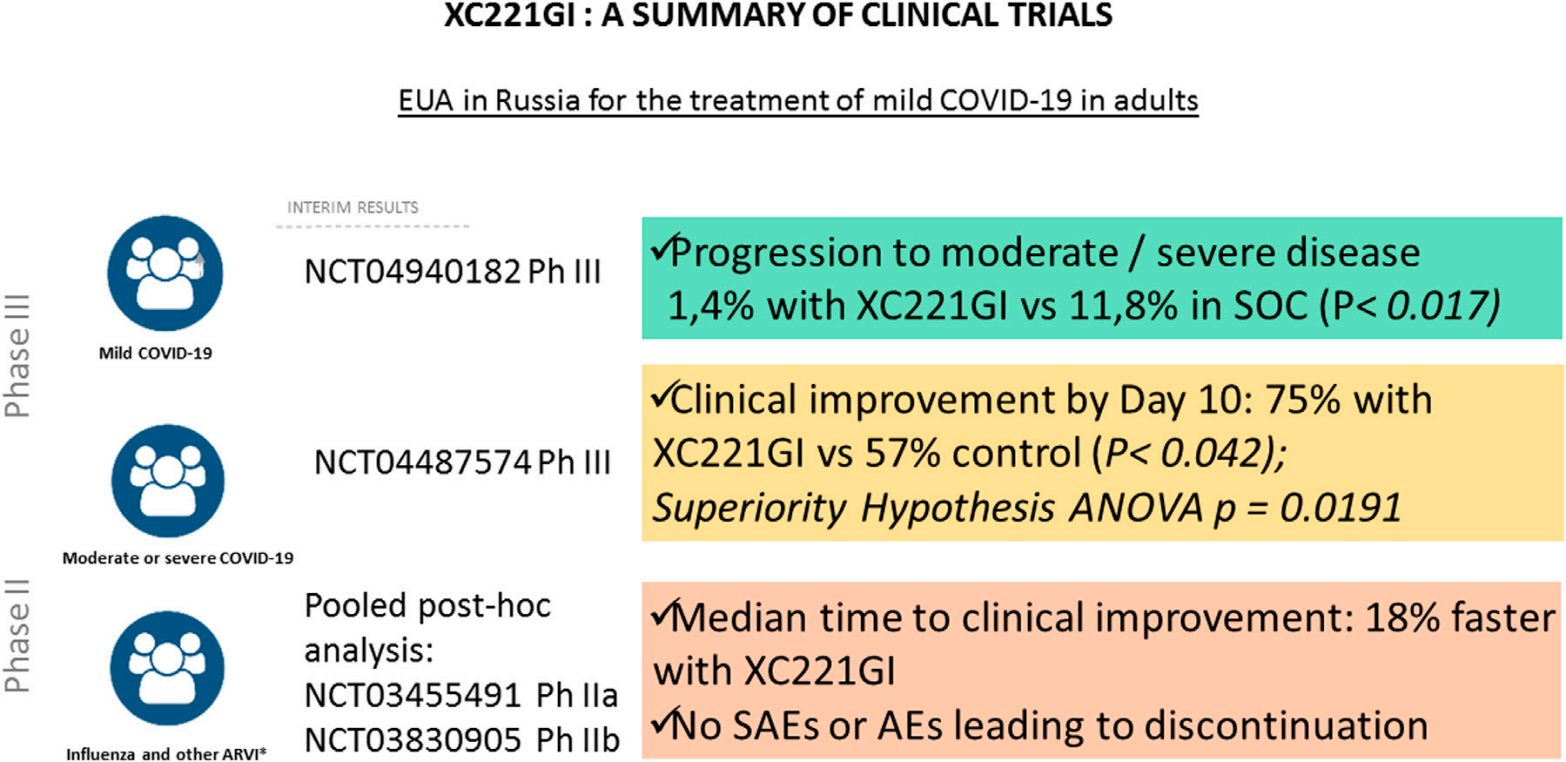
XC221GI: a summary of clinical trials.

In 2017–2022, a total of 7 clinical trials of XC221GI were completed. Its effects were tested in patients with influenza, acute viral upper respiratory infections, as well as moderate or severe COVID-19 with Phase I NCT03459391, Phase I XC221-01–04-2021, Phase IIa NCT03455491, Phase IIb NCT03830905, Phase II NCT05030324, Phase III NCT04487574 were completed (Figure 2).

In 2020, a multicenter, adaptive, randomized, double-blind, placebo-controlled phase III study was initiated to evaluate the efficacy and safety of XC221GI 100 mg tablet BID in hospitalized patients with COVID-19 (NCT04487574) with moderate or severe form of disease (t > 38.0°C; respiratory rate (RR) > 22/min; Sp02 < 95%) not requiring non-invasive/invasive ventilation and/or high-flow oxygenation (HFNT), and/or extracorporeal membrane oxygenation (EMO) at the time of screening and randomization). The main inclusion criteria were: age of 18–75 years, with COVID-19 diagnosis based on: 1) laboratory confirmation of the presence of the SARS-CoV-2 virus, carried out no earlier than 14 days before hospitalization and/or 2) bilateral changes in the lungs, characteristic of COVID-19. Two groups of patients were defined according to chest CT scan: moderate to severe COVID-19 (SpO2 ≥ 95%, T ˂ 38°C, RR ≤ 22/min, no lung damage according to CT scan), moderate to severe COVID-19 (t ≥ 38.0°C; RR ≥ 22/min; SpO2 < 95%) with oxygenation through a nasal cannula, a simple face mask or other similar oxygen delivery device with a score of 4 on the WHO scale. For both groups the first dose of the XC221GI drug was administered no later than seven full days from symptom manifestation.

Treatment Arm (n = 59) received XC221GI 100 mg BID for up to 14 days. Control arm (n = 59) received placebo for up to 14 days. Both groups received standard of care COVID-19 therapy according to the state guidelines for COVID-19 treatment (designed by the Ministry of Health of the Russian Federation).

The primary endpoint of the study was the proportion of patients with clinical improvement by Day 10 defined as all of the following: 1) body temperature ≤37.5 C without taking NSAIDs and/or acetaminophen; 2) RR ≤ 22/min without oxygen therapy; 3) SpO2 ≥ 95% without oxygen therapy.

A preliminary efficacy analysis for the primary endpoint at Day 10 in the Intention-To-Treat population demonstrated clinical improvement in 43 (75.4%) patients in the XC221GI Arm and in 34 (57.6%) patients in the Placebo Arm.

Analysis in the Per-Protocol (PP) population showed similar results: clinical improvement by Day 10 was observed in 77.8, and 59.6% of patients in the Treatment and Placebo Arm, respectively. Thus, both the main and additional analyses for the primary endpoint indicated superiority of the XC221GI over placebo (*p* = 0.0191). The proportion of patients who achieved clinical improvement in the XC221GI group was 1.3 times greater than that in the placebo group.

Notably, in the XC221GI Treatment Arm, the SpO_2_≥95% value was achieved almost a day faster than in Placebo Arm. Similarly, the treatment with XC221GI led to a faster decrease in body temperature, which was achieved by the evening of the second day. By the Day 6, the NEWS scores decreased to 2 or fewer points in 89.5% of patients in the XC221GI group and in 74.6% of patients in the placebo group. In the safety assessment of XC221GI, no significant differences between the Treatment and the Placebo Arms were detected (Gorelov et al., 2022).

Clinical findings of Phase III trial in a cohort of hospitalized patients with COVID-19 (NCT04487574) led to initiation of further Phase III NCT04940182 study in a cohort of ambulatory COVID-19 patients without established risk factors for the severe disease (ongoing). Meanwhile, in February 2022, XC221GI (Aterixen^®^) received marketing approval of the Ministry of Health of the Russian Federation under Emergency Use Authorisation. The results of the clinical studies of XC221GI are summarized on Figure 2 and Table 1.

## Discussion

The COVID-19 pandemic has exposed the shortcomings of current approaches to treating acute respiratory viral infections. Untreated COVID-19 has several overlapping phases, including the incubation period, the symptom development phase, the early inflammation phase, the secondary infection phase, and the multisystem inflammation phase (multisystem inflammation syndrome, MIS) [Griffin et al., 2021]. The incubation period can last from 2 to 14 days. In many cases, activation of innate immunity leads to rapid elimination of the virus and an asymptomatic form of the disease, which occurs in 40%–45% of cases (Oran and Topol, 2021; Long et al., 2020). With a less effective immune response, a host of symptoms appears, including fever, cough, muscle pain, loss of smell, and gastrointestinal problems. In elderly patients and in those with underlying medical conditions severe course of COVID-19 is common. Typically, the advert chain of events starts with underwhelming performance of unbalanced immune system, with low IFN type I production, overabundant IFN type II (γ) release and excessive secretion of IL6 and TNFα, known as “cytokine storm”. Untreated COVID-19 may also be complicated by a secondary bacterial or fungal infection requiring antibiotics and antifungals, and contributing to excessive immune response. Another common sequela of COVID-19 is organizing secondary pneumonia with damage to the bronchioles and alveoli, similar to observed in RSV infections and influenza (Brand et al., 2013). The lack of viral clearance, microvascular thrombosis, the tissue damage and opportunistic bacterial infections overburden patients (Renu et al., 2020), leading to hypoxemia, pulmonary disease, cardiac dysfunction, renal failure, neurological manifestations, and, ultimately, multiple organ failure (Mehta et al., 2020; Hadjadj et al., 2020), all of which are compounded by “cytokine storm” that perpetrates inflammatory insults and prevents return to homeostasis.

Current ways of dealing with the excessive inflammatory response (“cytokine storm”) are reactive rather than proactive, with corticosteroids accompanied by IL-6/JAK/STAT pathway inhibitors (baricitinib, tocilizumab, sarilumab) used to suppress the excessive immunological response, thus, preventing the sliding of patient into multi-organ failure (Sen et al., 2022). These medications are administered exclusively to patients with severe and critical COVID-19 symptoms, as the risk-benefit ratio of this therapy is favourable only for these groups. As a majority of COVID-19 do not display signs of severe illness at inception of their treatment, National and International guidelines have advised against preventative anti-inflammatory treatment with corticosteroids or IL-6/JAK/STAT inhibitors.

One way of preventing hyperinflammatory immune response is to apply direct anti-viral therapy. In case of COVID-19, two highly efficient compounds became available recently, including molnupiravir (REF) and nirmatelvir/ritonavir combination (REF). Unfortunately, global access to these medications remains limited [Usher, 2022], and a potential raise of DAAs-resistant variants is a concern (Horby et al., 2021). Because of that, the development of reliable drugs capable of systemic suppression of cytokine production is warranted (Malik et al., 2020; Azh et al., 2022). These anti-inflammatory drugs should be safer than corticoteroids, capable of early, preventative action, while not requiring molecular diagnostics of particular virus attacking the body. In this review, we have summarized available information on the anti-cytokine effects of XC221GI and its utility for preventing the development of the severe ARVIs in the patients predisposed to hyperiflammation.

**FIGURE 3:**
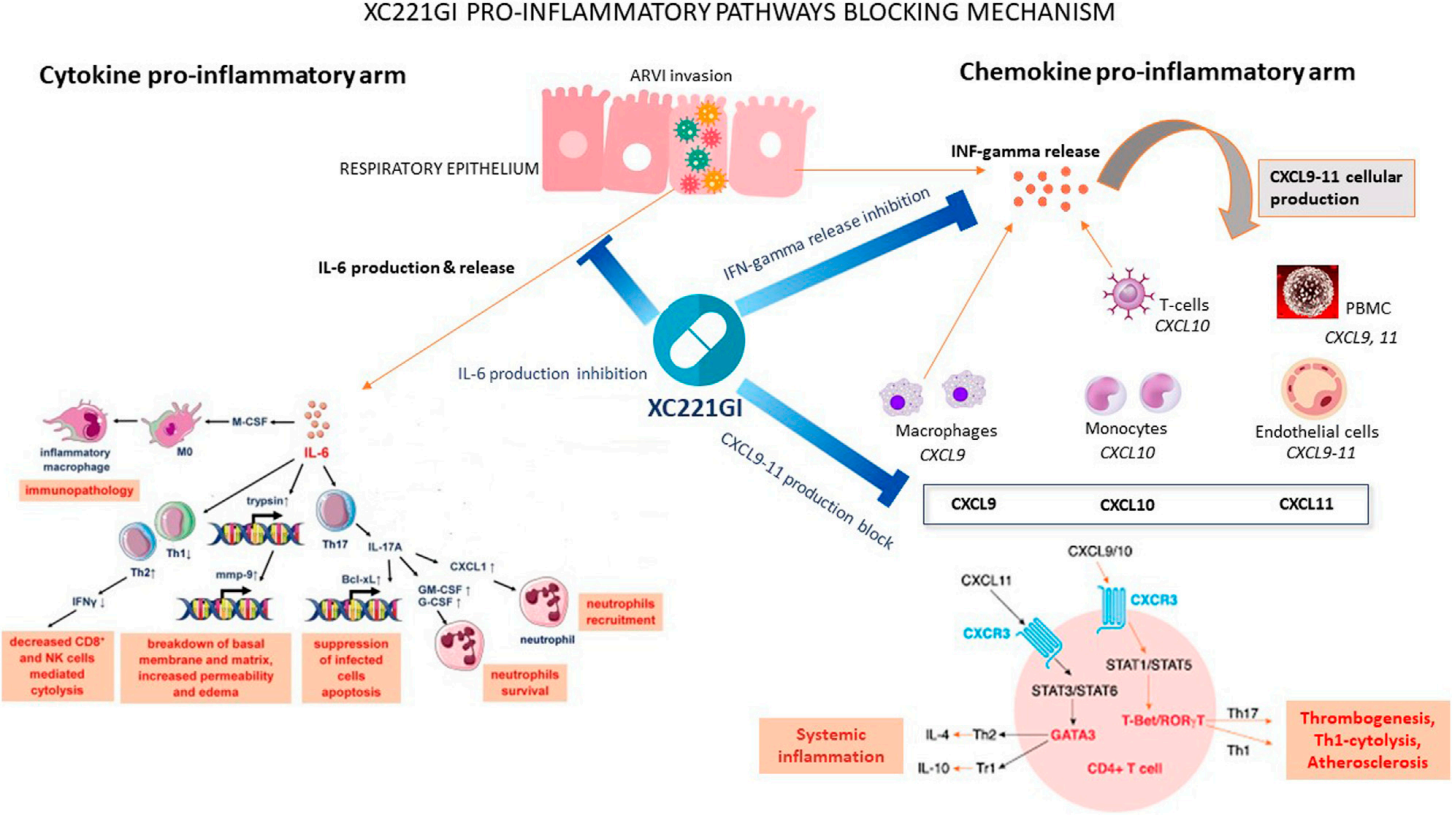
XC221GI pro-inflammatory pathways blocking mechanism.

In multiple animal models of respiratory diseases, the treatment with XC221GI led to controlling the levels of CXCL9, CXCL10, CXCL11, IL-6 and IL-8, and to decrease of extent of the pathogen-driven cytokine storm. Notably, while the properties of TNF-alpha, IL-6 and IL-8 as hyperinflammatory drivers are well known [Vardhana and Wolchok, 2020; Sinha et al., 2020], the ligands of chemokine receptor CXCR3 chemokine receptor ligands, namely, CXCL10 (IP-10), CXCL11 (I-TAC), and CXCL9 (MIG), are deserving more detailed attention (Landry and Foxman, 2018; Hayney et al., 2017; Van Raemdonck et al., 2015; Matsuda et al., 2014). These chemokines are produced by epithelial cells, monocytes, endothelial cells, and smooth muscle cells of the airways when stimulated by IFNγ, type I interferons, and tumor necrosis factor TNFα. Serving as chemokines, CXCL9, CXCL10, and CXCL11 attract the cells with their cognate receptor CXCR3, including neutrophils, monocytes/macrophages, dendritic cells, T cells, and NK cells, to the site of infection (Callahan et al., 2021). When this chemokine trail is curtailed, migration of effector cells to the site of infection is halted. Thus, no massive, unbalanced inflammation would ensue. The mechanisms of action of XC221 are summarized in Figure 3.

Specifically in COVID-19, the CXCL10-CXCR3 axis serves as a critical factor in developing pulmonary pathology, including tissue edema and microvascular microthrombosis (Ichikawa et al., 2013; Callahan et al., 2021). In particular, increased expression of CXCR3-binding chemokines was found in bronchoalveolar lavage (BAL) and peripheral blood mononuclear cells (PBMC) of patients with severe COVID-19, while CXCR3-bearing neutrophils were found in pulmonary tissue in COVID-19 (Rossi and Zlotnik 2000), but not in healthy individuals (Hartl et al., 2008). Similarly to COVID-19, in other ARVIs, the levels of IFNγ-inducible CXCL10 in BAL and plasma correlate with the severity of the disease (Hayney et al., 2017; Almansa et al., 2011; Buszko et al., 2020). It is also important to note that the levels of individual proinflammatory cytokines and chemokines commonly co-correlate, closely following underlining pathophysiological process and reflecting its intensity (Sinha et al., 2020; Tabarani et al., 2013; Brand et al., 2013; Jafri et al., 2004). In addition, IL8 plays a vital role in developing the prothrombotic neutrophil phenotypes. Blocking IL8 signaling with an anti-IL8 monoclonal antibody or a CXCR1/2 receptor blocker (reparixin) (Opfermann et al., 2015) reduced both neutrophil activation *in vitro* and the severity of acute respiratory distress syndrome (ARDS) caused by SARS -CoV-2 *in vivo*.

One of the most common ways to decrease intensity of inflammation is to suppress the signal emitted by most prominent components of cytokine storm, namely, TNF-alpha and IL-6. For example, monoclonal antibodies tocilizumab and sarilumab antogonize the IL-6 receptor itself, while Jak inhibitors baricitinib and tofacitinib work downstream of this cytokine-binding signalling event by inhibiting intracellular steps of the same signal transduction (Bhimraj et al., 2022). While these therapeutic approaches are very powerful from the molecular point of view, their effects on the patients with severe forms of COVID-19 are rather incremental. For example, in COVID-19 patients having clinical progression despite the therapy with tocilizumab and corticosteroids, adding baricitinib did not substantially reduce mortality (Masia et al., 2021). Other studies show that tocilizumab does not provide benefit to moderately ill hospitalized patients with COVID-19 at risk of intubation or death (Stone JH et al., 2020). The failure of anti-IL6 therapy to produce major therapeutic effect in COVID-19 was recently explained by Jiang et al., who showed IL-6 interaction with the signalling glycoprotein gp-130 on the surface of T cells may occur with no involvement of IL-6R (Jiang Z et al., 2021). As the CXCR3-binding chemokines work upstream of IL-6, curtailment of this inflammatory signal provides an attractive avenue for the preventing rather than the extinguishing of the cytokine storm. In this light, it is important to note that in epithelial cells infected with RSV, XC221GI effectively suppressed RSV-induced production of IL-6 (45%–85%) and IL-8 (40%), while the steady-state, spontaneous secretion of these cytokines was not affected (Stukova et al., 2022). Thus, suppressing upstream elements of inflammatory cascade with XC221GI spreads onto downstream elements of the same regulatory pathway, thus, providing a systemic effect.

In animals exposed to IFNγ, the treatment with XC221GI significantly suppressed the influx of neutrophils into the bronchoalveolar space of the lungs. These effects were concomitant with a decrease in the production of all three ligands, CXCL9, CXCL10, and CXCL11, which was noticeable as early as 8–12 h after the administration of interferon. The scale of observed XC221GI effects on the influx of neutrophils was similar to that of dexamethasone 1 mg/kg. Notably, the treatment with dexamethasone affected only the levels of CXCL9, while in case of the treatment with XC221GI the levels all three chemokines were suppressed (Stukova et al., 2022).

Taken together, these results suggest that XC221GI may find its utility as a preventive anti-inflammatory therapy of the respiratory infections in the cohorts at risk of hospitalization, especially when rapid diagnostics of the viral species is impossible or when direct antiviral drugs are not available or are not indicated. After favourable assessment of its safety profile, XC221GI was advanced to Phase II and Phase III trials in patients with influenza, with unclassified acute viral upper respiratory infections, and with COVID-19. In 2022 XC221GI received Emergency Use Authorization in Russia for treating mild cases of COVID-19 in adults. As no head-to-head trials of XC221GI were run to compare its effects with that of direct antivirals, the clinical efficacy parameters described so far should be interpreted with caution. Nevertheless, an excellent safety profile and significant anti-inflammatory effects of XC221GI indicate its utility in abating conversion of ambulatory cases of respiratory infections into the cases with aggravated presentation that require hospitalization.
